# Molecular markers associated with cognitive impairment in centenarians

**DOI:** 10.18632/aging.204094

**Published:** 2022-05-18

**Authors:** Tinna Stevnsner, Ines Sanchez-Roman

**Affiliations:** 1 Department of Molecular Biology and Genetics, Aarhus University, Aarhus, Denmark; 2 Danish Aging Research Center, Aarhus, Denmark; 3 Department of Genetics, Physiology and Microbiology, Faculty of Biological Sciences, Animal Physiology Unit, School of Biology, Complutense University of Madrid, Madrid, Spain

**Keywords:** centenarians, cognition, DNA maintenance, mitochondria, oxidative stress

Aging is strongly associated with cognitive decline and degenerative diseases. Therefore, unravelling the basic mechanisms of aging is essential to improve the quality of life of older people. Among others, mitochondrial dysfunction and genomic instability have been considered key hallmarks of aging, and these deficiencies seem to play important roles in age associated cognitive decline [1]. Mitochondrial bioenergetic deterioration together with reactive oxygen species (ROS) induced mitochondrial DNA damage have been shown to accumulate with aging [2]. DNA repair pathways, which are involved in maintaining genomic stability, also seem to change with aging. Most oxidative DNA lesions are repaired by the Base Excision Repair (BER) pathway, in which the major endonuclease at the limiting step is AP endonuclease 1 (APE1), which has also been linked to cognitive decline [3]. Mitochondrial function and DNA repair activity are affected by nicotinamide adenine dinucleotide (NAD) depletion, which is also observed with aging. NAD is an essential co-enzyme involved in mitochondrial health and functions as a cofactor for the DNA repair protein poly (ADP-ribose) polymerase 1 (PARP-1) [4]. Another factor that has been shown to decline with aging and be reduced in neurodegenerative diseases is brain derived neurotrophic factor (BDNF). Importantly, BDNF has been shown to enhance neuronal DNA repair by stimulating APE1 expression and activity [5].

Although the role of the factors mentioned above has been extensively studied, it is still not known whether these aging related factors can serve as indicators of cognitive function in human studies. In a recent paper, we have addressed this question and performed molecular analyses on blood cells and plasma with the purpose of identifying key biomarkers related to cognitive abilities in very old individuals [6]. We performed our studies on a cohort of centenarians, a selective population with unique features. This particular group is valuable as a model of healthy aging and represents a wide heterogeneity in regard to cognitive capacities.

We used Seahorse real-time cell metabolic analysis technology to measure different mitochondrial parameters in peripheral blood mononuclear cells (PBMCs). Centenarians were found to display well-preserved mitochondrial respiration in their PBMCs when compared to young individuals. Additionally, we observed that only centenarian males and not females showed a negative correlation between the rate of mitochondrial respiration and cognitive scores. Thus, our results suggest that lower rates of mitochondrial respiration may indicate better cognitive capacities in males. This agrees with a recent study performed in mice, which also associated a moderation of the mitochondrial activity with better health outcomes [7]. We believe that an unbalance in the regulation of the mitochondrial physiology (e.g.; mitochondrial biogenesis and mitophagy among others) could lead to a change in mitochondrial activity, that may be translated into increased oxidative stress and cellular deterioration.

Our study revealed that sex differences must be considered as an important biological variable in research within the aging field. We have found negative correlations between cognitive abilities and mitochondrial respiration only in males, which could be due to the sexual dimorphism that has been observed for mitochondrial function, in the way females and males cope with oxidative stress [8]. Although there is extensive research in the field of aging, traditionally the sex of experimental subjects has not been balanced, and there is relatively limited information on whether biological aging presents differently in men and women. Therefore, this needs to be revisited in future studies.

Furthermore, we found that an increased amount and activity of the core BER enzyme, APE1 in PBMCs correlated with well-maintained cognitive health in both sexes. In the same way, the amount of circulating BDNF and NAD^+^/NADH levels in blood plasma, also correlated positively with cognitive capacity. Lastly, our results showed a high level of protein carbonylation, which is an indicator of oxidative stress, in individuals with lower cognitive scores. Based on these observations, we hypothesize that increased NAD^+^/NADH levels as well as BDNF could possibly slow down cognitive decline or aging related diseases through the stimulation of DNA repair pathways and the consequent lowered oxidative stress. This finding incites to look deeper into the network formed by DNA damage sensing proteins, DNA repair pathways and oxidative stress in relation to aging. Of note, NAD+ boosting strategies are already under investigation in order to prevent the loss of cognitive function in humans [4].

**Figure 1:**
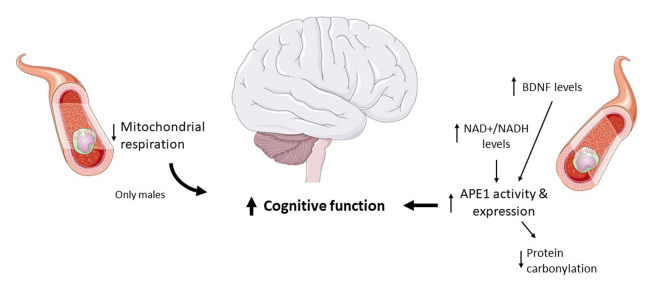
**Molecular markers associated with maintenance of cognitive capacity in centenarians.** The figure was generated using pictures from Servier Medical Art, provided by Servier, licensed under a Creative Commons Attribution 3.0 unported license.

In summary, we have presented several molecular markers that can be measured in blood and which correlate with the maintenance of cognitive capacities. However, in order to be able to design strategies to delay the aging associated cognitive impairment in humans, we still need a better understanding of the interplay between factors like NAD^+^, BDNF, DNA repair and mitochondrial function in relation to aging and cognitive decline (Figure 1).
